# Guiding locomotion in complex, dynamic environments

**DOI:** 10.3389/fnbeh.2013.00085

**Published:** 2013-07-19

**Authors:** Brett R. Fajen

**Affiliations:** Department of Cognitive Science, Rensselaer Polytechnic InstituteTroy, NY, USA

**Keywords:** locomotion, optic flow, obstacle avoidance, object motion perception, affordance perception

## Abstract

Locomotion in complex, dynamic environments is an integral part of many daily activities, including walking in crowded spaces, driving on busy roadways, and playing sports. Many of the tasks that humans perform in such environments involve interactions with moving objects—that is, they require people to coordinate their own movement with the movements of other objects. A widely adopted framework for research on the detection, avoidance, and interception of moving objects is the *bearing angle model*, according to which observers move so as to keep the bearing angle of the object constant for interception and varying for obstacle avoidance. The bearing angle model offers a simple, parsimonious account of visual control but has several significant limitations and does not easily scale up to more complex tasks. In this paper, I introduce an alternative account of how humans choose actions and guide locomotion in the presence of moving objects. I show how the new approach addresses the limitations of the bearing angle model and accounts for a variety of behaviors involving moving objects, including (1) choosing whether to pass in front of or behind a moving obstacle, (2) perceiving whether a gap between a pair of moving obstacles is passable, (3) avoiding a collision while passing through single or multiple lanes of traffic, (4) coordinating speed and direction of locomotion during interception, (5) simultaneously intercepting a moving target while avoiding a stationary or moving obstacle, and (6) knowing whether to abandon the chase of a moving target. I also summarize data from recent studies that support the new approach.

## Introduction

Getting from point A to point B is typically not simply a matter of moving along a straight path through a static environment over a flat, obstacle-free ground surface. More often than not, the environment contains moving objects, obstacles that must be stepped over, avoided, or dodged, and surfaces that vary in traction, slant, extent, and compliance. In such situations, vision plays a central role in allowing us to follow safe and efficient routes to the goal (Patla, 1997).

One of the challenges of developing a general theory of locomotion in complex environments is understanding how people coordinate their own movements with the movements of other objects. Moving objects can be either targets to be intercepted or obstacles to be avoided. One approach to this problem is to begin with the simplest forms of interception and obstacle avoidance and build up to more complex tasks. Indeed, it is not uncommon in studies on interception for the target to be the only object in the environment and to be moving slow enough to be easily catchable, and for either speed or direction of observer locomotion to be held constant (e.g., Lenoir et al., 1999; Chardenon et al., 2002; Bastin et al., 2006; Diaz et al., 2009).

However, many environments contain multiple moving objects. In such situations, aspects of locomotor control that are not particularly relevant in simple environments become critical in more complex environments. For example, it is easy to overlook the significance of one's body dimensions and locomotor capabilities in a single-target interception task with a slow-moving target. However, when there are also stationary and moving obstacles in the environment, the dimensions of one's body and other objects cannot be ignored. Similarly, if there is a possibility that the target is too fast to intercept, then the decision about whether to pursue the target in the first place must be made in a way that takes one's locomotor capabilities into account.

Theories and models based on interception and obstacle avoidance in simple, idealized situations do not easily scale up to more complex tasks because they often neglect aspects of locomotor control that are only important in more complex environments. In this paper, I introduce a new theory of locomotion in complex dynamic environments that was developed from the beginning to capture these important aspects of behavior and to apply to a wider range of tasks involving multiple moving objects.

## The bearing angle model

### Description of the bearing angle model

**Figure 1:**
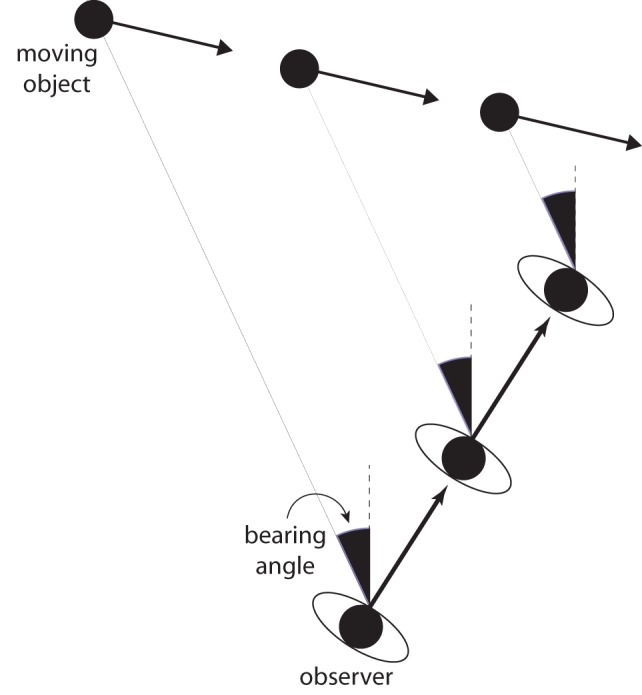
**The bearing angle model of interception and obstacle avoidance.** The bearing angle is the angle between the object and reference direction that remains fixed in exocentric coordinates (dashed line). By keeping the bearing angle constant, the observer will eventually intercept the moving target.

Before introducing the new theory, I will describe the *bearing angle* (BA) model, which is a widely adopted model of collision detection, obstacle avoidance, and interception. The BA model is based on a simple heuristic that is well-known to pilots and sailors for detecting imminent collisions. If an object's bearing (i.e., its direction relative to some fixed reference direction) remains constant, the observer and the object are on a collision course (Figure 1). If the object is an obstacle to be avoided, then a fixed bearing angle specifies that evasive action is called for. The observer should change speed or direction (or both) to avoid a collision. If the object is a target to be intercepted, and if the bearing angle is decreasing, then the observer's speed is insufficient for his or her direction of travel. To intercept the target, the observer should accelerate, turn ahead of the target, or both. Similarly, if the bearing angle is increasing, then the observer should decelerate, turn toward the target, or both.

The BA model is widely used to account for collision detection, interception, and obstacle avoidance in both humans (Cutting et al., 1995; Lenoir et al., 1999, 2002; Chardenon et al., 2004; Fajen and Warren, 2004; Ni and Andersen, 2008; Shaffer and Gregory, 2009) and non-human animals (Lanchester and Mark, 1975; Olberg et al., 2000; Ghose et al., 2006). In addition, the behavioral dynamics model (Fajen and Warren, 2003; Warren, 2006), which is one of the few general models of visually guided locomotion in humans, implements the BA strategy in its moving target (Fajen and Warren, 2007) and moving obstacle (Cohen et al., 2006) components.

### Limitations of the BA model

Although the BA model provides a parsimonious account of some aspects of interception and obstacle avoidance, the model has a number of significant limitations:

#### Limitation #1

The BA model treats the observer's body and objects as points without any physical extent. This is especially problematic for obstacle avoidance, where the physical size of the observer's body and the obstacles must be taken into account to avoid collisions.

#### Limitation #2

The BA model ignores the fact that there are limits to how fast actors can move and how quickly they can turn. Such limits must be taken into account to choose appropriate actions and to properly guide locomotion (Fajen, 2005a, 2007). For example, when crossing the street, the decision to go ahead of the approaching vehicle or wait until after the vehicle passes must be made in a way that takes one's locomotor capabilities into account. Because the BA model ignores the fact that such limits exist, it provides no explanation of how actions are selected, initiated, and guided in a way that takes locomotor capabilities into account.

#### Limitation #3

Most real-world interception and obstacle avoidance tasks are minimally two degree-of-freedom control tasks in that actors can change both direction and speed of locomotion. The BA model provides no explanation of how these two degrees of freedom are coordinated (Bastin et al., 2010). A decrease in the bearing angle specifies that the observer's current speed (given his or her current direction) is insufficient, and that the observer should increase speed, turn ahead of the target, or both. However, the model does not provide a basis for understanding how observers decide when to change speed only, when to change direction only, and when to change both speed and direction.

#### Limitation #4

The BA model is incompatible with findings demonstrating that behavior is influenced by manipulations of self-motion information. According to the BA model, locomotion is guided by the change in bearing angle of the object; that is, by the local optical motion of the moving object, independent of the global optical motion corresponding to other, stationary features of the environment such as the ground plane. Therefore, manipulations of optic flow originating from the stationary background should not influence the observer's behavior during interception or obstacle avoidance. Contrary to this prediction, when people walk to intercept a moving target or choose routes around moving obstacles, manipulations of the background optic flow do influence behavior (Fajen and Warren, 2004; Fajen and Matthis, 2013).

To summarize, the BA model offers a simple, elegant account of certain aspects of interception and obstacle avoidance, but has several significant limitations. One can begin to appreciate the consequences of these limitations by considering a task such as walking across a busy street. According to the BA model, the observer should move so as to ensure that the bearing angle of each individual moving obstacle (e.g., vehicles, bicycles, other pedestrians) does not remain constant. The model offers no account of how people decide when to initiate locomotion, how they take their physical size and the sizes of obstacles into account, how they take their locomotor capabilities into account, or how they coordinate speed and direction of locomotion.

## The affordance-based model

In this section, I will introduce a fundamentally different account of how locomotion is controlled in the presence of other moving objects, and explain how this approach offers a better starting point for addressing the weaknesses of the BA model. The guiding principle of the new approach is that observers choose actions and control locomotion in a way that takes their body dimensions and locomotor capabilities into account (Fajen, 2007). This begins with the perception of *affordances*—that is, possibilities for action provided by the environment for an observer with particular dimensions and capabilities (Gibson, 1986; Turvey, 1992; Fajen et al., 2008). As such, the new approach will be referred to as the *affordance-based model*. The task of avoiding a single moving obstacle will be used to illustrate the basic concepts of this new approach. In the following section, I will explain why this approach is better suited for more complex tasks involving moving objects.

### Model assumptions

The affordance-based model applies in situations in which the following assumptions are satisfied. First, the observer and objects are in contact with a flat, level ground surface. In its present form, the model does not apply in situations in which objects are moving through air, and therefore does not currently account for the interception of projectiles (e.g., a fly ball in baseball) or prey catching in fish or flying insects. Second, the model assumes that targets and obstacles move at a constant velocity. The model will still function if objects change speeds or directions, but it does not in its current form account for observers' ability to anticipate predictable changes in velocity (Diaz et al., 2009). Third, the model assumes that the relation between the observer's eyeheight (*E*) and body width (*W*) is stable. In the event that either eyeheight or body width changes, it would be necessary for the perceptual-motor system to recalibrate itself to this change. Previous research has demonstrated that recalibration to changes in eyeheight occurs rapidly and with minimal movement (Mark et al., 1990).

### Body-scaled information

The affordance-based model is based on *body-scaled visual information* (Lee, 1980; see Warren, 2007 for a recent review) that specifies dimensions of the environment in relation to dimensions of the observer. The hypothesis that certain dimensions of the environment (e.g., object size) are perceived on the basis of body-scaled information is supported by studies demonstrating that subtle manipulations of eyeheight influence perception of the environment (Mark, 1987; Warren and Whang, 1987; Wraga, 1999).

**Figure 2:**
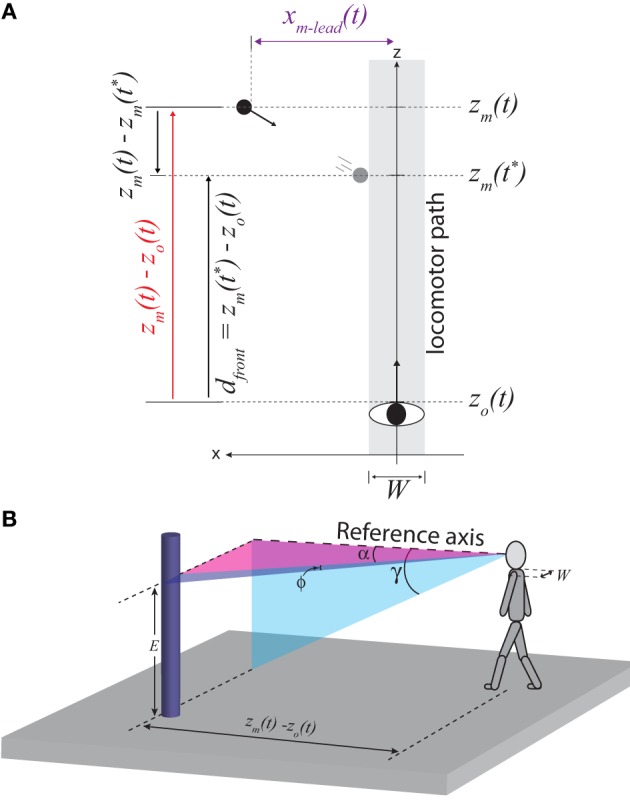
**(A)** Top-down view of observer (body width, *W*) moving straight ahead and an object (black circle) moving from left to right. *z* and *x* correspond to the positions of the observer (subscript o) and moving object (subscript *m*), respectively. *t* corresponds to the current time and t^*^ corresponds to the time at which the leading edge of the obstacle reaches the left side of the locomotor path. **(B)** Illustration of optical angles used in Equations 1–8.

Figure 2A depicts an object moving from left to right across the observer's future path. I will consider four properties related to the position and movement of the object in Figure 2A and the specification of these properties by body-scaled information. For the reader's convenience, definitions of the main spatial, temporal, and optical variables used in the equations below are provided not only in the text but also in Table 1.

**Table 1:** **Definitions of symbols used to designate spatial, temporal, and optical variables**.

**Symbol**	**Definition**	**Figures**
**SPATIAL AND TEMPORAL VARIABLES**		
*x_o_, z_o_*	Position of observer along *x* and *z* axes, respectively	2A
*x_m_, z_m_*	Position of moving object along *x* and *z* axes, respectively	2A
*W*	Width of observer's body	2A
*E*	Observer's eyeheight	2B
V_max_	Maximum speed at which the observer is capable of moving	–
*t*	Current time	–
t^*^	Time at moment that leading edge of object first reaches the locomotor path (gray area in Figure 2A)	–
TTC	Amount of time remaining until the object crosses the reference axis	–
*k*	A parameter equal to one-half of the ratio of the observer's body width (*W*) to his or her eyeheight (*E*)	–
*d*_front_	Minimum distance that the observer must travel to pass in front of the moving object	2A
*d*_behind_	Minimum distance that the observer must travel to pass behind the moving object	–
*v*_front_	Minimum speed at which the observer must travel to pass in front of the moving object	–
*v*_behind_	Maximum speed at which the observer could travel and still safely pass behind the moving object	–
**OPTICAL VARIABLES**		
α	Angle between reference direction and moving object	2B
θ	Angle subtended by edges of moving object	2B
γ	Angle of declination of projection of base of object along reference axis	2B

#### Distance along z-axis

In terms of spatial variables, the distance to the object along the z-axis at time *t* is equal to *z_m_(t)* – *z_o_(t)*, where *z_m_* and *z_o_* are the positions of the moving object and observer along the z-axis, respectively (see Figure 2A). *z_m_(t)* – *z_o_(t)* is optically specified in units of the observer's eyeheight (*E*) by:

(1) $$\left[z_{m}(t)-z_{o}(t)\right]/E=1/\text{​}\tan\gamma$$

where γ is the angular declination of the base of the object along the z-axis (see Figure 2B)^1^. This is based on the well-known depth cue *height in the visual field*.

#### Lateral position

The lateral position of the leading edge of the moving object (*x_m_*_− lead_) is optically specified in units of *E* by:

(2) $$\left[x_{m\;-\text{ lead}}(t)\right]/E=\tan\;\alpha /\tan\;\gamma$$

where α is the optical angle between the reference axis and the leading edge of the object (adapted from Warren and Whang, 1987; see Figure 2B).

#### Approach speed

The approach speed of the object along the z-axis $\left[-{\dot{z}}_{m}(t)\right]$ is optically specified in units of *E* by:

(3) $$\left[-{\dot{z}}_{m}(t)\right]\text{​}/E={\dot{\gamma }}_{m}/\sin^{2}\gamma$$

where $\dot{\gamma }$ is the rate of change of γ and the subscript *m* designates the component of $\dot{\gamma }$ that is due to the motion of the moving object independent of the motion of the observer [adapted from Lee (1980)]. When both the observer and object are in motion, $\dot{\gamma }$ is the sum of two components: ${\dot{\gamma }}_{o}$, which is the rate of change of γ due to the observer's self-motion, and ${\dot{\gamma }}_{m}$, which is the rate of change of γ due to the motion of the object. This means that detecting information about $-{\dot{z}}_{m}(t)$ requires the visual system to recover the component of $\dot{\gamma }$ that is due to observer motion. As I will explain below, this has important implications for the detection of information.

#### Time-to-crossing

The amount of time remaining until the leading edge of the object crosses the z-axis is optically specified by:

(4) $$TTC={\left(\frac{\dot{\phi }}{\sin\phi \;}-\frac{\dot{\alpha }}{\tan\alpha \;}\right)}^{-1}$$

where ϕ is the visual angle subtended by the edges of the object (Bootsma and Craig, 2002), as illustrated in Figure 2B^2^.

Next, I will show how the availability of information about distance along the z-axis, lateral position, approach speed, and TTC make it possible for people to perceive higher-order properties that are directly relevant to guiding interception and obstacle avoidance. In terms of spatial variables, the minimum distance that the observer would need to move along the z-axis to safely pass in front of the obstacle (*d*_front_) at time *t* is:

(5) $$d_{\text{front}}(t)=z_{m}\text{​}\left(t^{*}\right)-z_{o}(t)$$

where *z_m_* and *z_o_* are the positions along the z-axis of the moving obstacle and the observer, respectively, and t^*^ is the time at which the leading edge of the obstacle reaches the left side of the locomotor path (i.e., the gray region in Figure 2A aligned with the z-axis of width equal to the observer's body width, *W*). The subscript “front” is used to indicate the distance needed to pass in front of the obstacle. Equation 5 can be rewritten as:

(6) $$\frac{d_{\text{front}}(t)}{E}=\frac{\left[z_{m}(t)-z_{o}(t)\right]}{E}+\frac{{\dot{z}}_{m}}{E}\times TTC\times \left[1-k\left[\frac{E}{x_{m\;-\text{ lead}}(t)}\right]\;\right]$$

where *k* is a constant equal to one-half of the ratio of the observer's body width (*W*) to his or her eyeheight (see Appendix for derivation of Equation 6).

In Equation 6, *d*_front_*/E* is expressed in spatial variables, which are not directly accessible to the perceptual system. As shown in Equation 7, however, each individual component of Equation 6 can be expressed in terms of optical variables using Equations 1–4: 
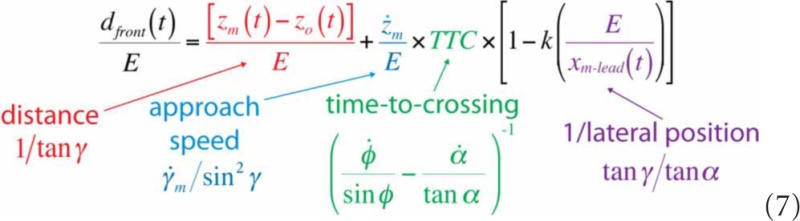


Equation 7 shows that there is information in the optic array that specifies the minimum distance that the observer would need to move to safely pass in front of the obstacle (*d*_front_), taking into account the physical size of both his or her body and the obstacle. Similarly, the amount of time remaining before the leading edge of the obstacle reaches the locomotor path (i.e., *t_front_* = t^*^ − *t*) is also optically specified: 
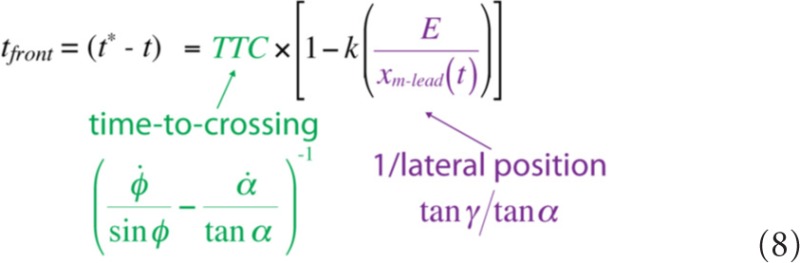


Thus, information is available that allows observers to perceive how far to move to pass in front (Equation 7), how much time remains before it is no longer possible to pass in front (Equation 8), and the ratio of these two quantities, which is the minimum locomotor speed needed to pass in front (*v*_front_).

The abovementioned information specifies properties for passing in front of the obstacle. Observers must also perceive properties relevant for passing behind the obstacle: the distance they need to travel to pass behind (*d*_behind_), the amount of time remaining until the trailing edge of the obstacle reaches the far side of the locomotor path (*t*_behind_), and the maximum speed that the observer could move and still pass behind (*v*_behind_). Distance to pass behind is specified by a variant of Equation 7 that uses the angular azimuth of the trailing (rather than leading) edge of the obstacle, and adds *k×(E/x_m_*_−trail_) rather than subtracts *k×(E/x_m_*_−lead_). With the same modifications, Equation 8 specifies the amount of time remaining until the trailing edge passes the far side of the locomotor path. The ratio of these two quantities is equal to the maximum speed at which the observer could move and still pass behind (i.e., *v*_behind_ = *d*_behind_/*t*_behind_).

**Figure 3:**
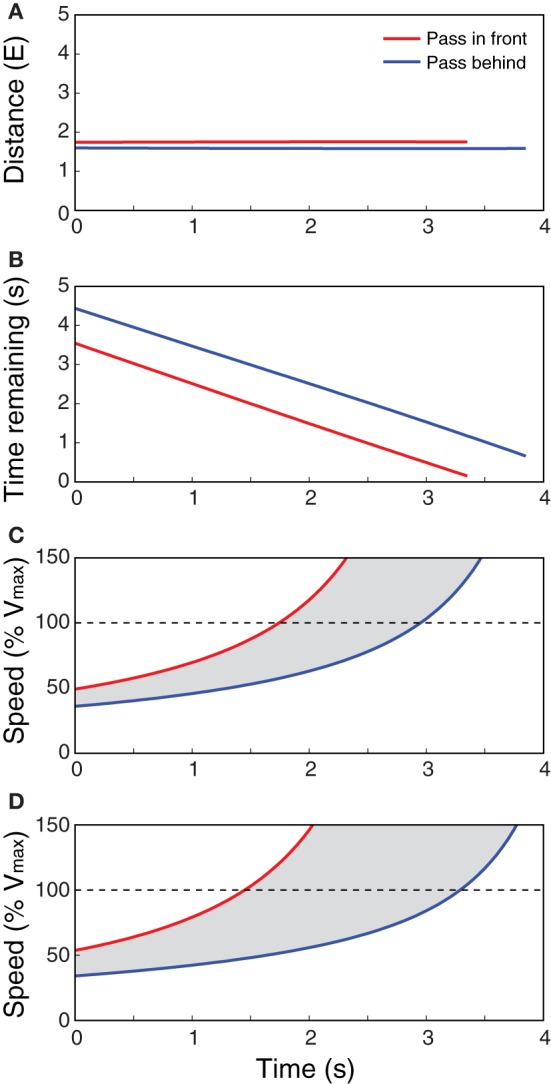
**Optically specified distance (A), time remaining (B), and speed (C,D) to pass in front (red line) and behind (blue line) for a stationary observer and a moving obstacle, similar to the situation depicted in Figure 2A. (A–C)** Are for a small obstacle, and **(D)** is for a larger obstacle. Values were calculated using optical variables in Equations 7 and 8. *E* is the observer's eyeheight, s is seconds, and *V*_max_ is the observer's maximum locomotor speed. Gray area in **(C)** and **(D)** indicate range of speeds that would result in a collision.

Taken together, the available information specifies the minimum speed needed to pass in front (*v*_front_) and the maximum speed to pass behind (*v*_behind_), or equivalently, the range of speeds that would result in a collision between some part of the observer's body and some part of the obstacle. Figures 3A–C shows the optically specified values of distance, time remaining, and required speed for passing in front and passing behind as a function of time for a stationary observer and a moving obstacle, similar to the situation depicted in Figure 2A. The values were calculated using the optical variables in Equations 7 and 8.

### Taking body size and object size into account

Recall that one of the weaknesses of the BA model is that it treats objects and the observer as points (see Limitation #1), which means that it does not explain how observers take their body size or the sizes of objects into account. In this section, I will explain how the affordance-based model addresses this problem.

To pass in front of a moving obstacle, an observer with a large body size must travel farther and cover that distance in less time than an observer with a small body size. Indeed, Equations 7 and 8 yield values of *d*_front_/*E* and *t*_front_ that take observer size into account in exactly this manner. This is because the observer's size is implicitly represented by the parameter *k*, which is equal to one-half of the observer's body width (*W*) in units of eyeheight (*E*); that is, *k* = 1/2 × *W/E*.

A cognitive interpretation is that *k* represents the observer's knowledge of his or her body size and eyeheight. However, proponents of non-representational accounts of visually guided action can take comfort in the fact that *k* is in fact the *relation* between body size and eyeheight. It is body size in units of eyeheight (i.e., in intrinsic units). The observer need not know either body size or eyeheight in absolute, extrinsic units, but merely the relation between these dimensions. Therefore, *k* is arguably better construed as a unit-free parameter that is learned through active exploration. The outcome of such learning is a properly calibrated perceptual system that allows for the accurate perception of *d*_front_/*E* and *t*_front_ (see Bingham and Pagano, 1998; Fajen, 2005b; Jacobs and Michaels, 2006 for similar accounts involving calibration). From this perspective, the only sense in which the observer “knows” his or her body size is in terms of the state of calibration of the perceptual system.

The relevant properties are also specified in a way that takes into account the size of the obstacle. This is illustrated in Figures 3C,D, which shows the optically specified values of *v*_front_ and *v*_behind_ for a smaller obstacle (Figure 3C) and a larger obstacle (Figure 3D) moving at the same speed along the same trajectory. Note that the information specifies how much faster the observer would have to move to pass in front of the larger obstacle and how much slower he or she would have to move to pass behind. Thus, by detecting this information, observers can choose routes and guide locomotion in a way that takes observer and object size into account. This addresses Limitation #1 of the BA model.

### Taking locomotor capabilities into account

The affordance-based model also differs from the BA model in that it provides a basis for understanding how people take their locomotor capabilities into account. To illustrate this point, let us suppose that the observer and the obstacle in Figure 2A are moving in such a way that they would collide if both maintain their current velocity. If the observer is in a hurry, then she may choose to speed up to pass in front of the obstacle. However, if the speed needed to pass in front is faster than the speed that the observer is capable of moving, then the act of passing in front has no chance of succeeding. Accelerating in an attempt to pass in front would result in wasted energy or worse, a collision. The sooner the observer perceives that it is not within her locomotor capabilities to pass in front, the better. To decide whether to pass in front or pass behind, the observer must perceive the minimum speed needed to pass in front (*v*_front_) in relation to the maximum speed that the observer is capable of moving (*V*_max_).

Again, there is a cognitive interpretation that treats *V*_max_ as knowledge of one's maximum locomotor capabilities. By this account, the decision about whether to pass in front or pass behind involves a comparison of a perceived quantity (*v*_front_) to a known quantity (*V*_max_). However, the fact that *v*_front_ is specified in units of eyeheight opens the door to a less cognitive interpretation. During locomotion over a solid support surface, observers' eyeheight (*E*) and maximum locomotor speed (*V*_max_) remain relatively stable. Therefore, the relation between these two quantities remains stable. As such, the fact that *v*_front_*/E* is optically specified means that *v*_front_*/V*_max_ is also optically specified. The logic of this argument is the same as that used by Warren and Whang (1987), captured in the following quote, to claim that eyeheight-scaled information about the size of an aperture specifies aperture size in relation to shoulder width: “Because standing eyeheight (*E*) bears a constant relation to shoulder width (*W*) in any individual, optical information specifying the ratio *A/E* also provides information about the ratio *A/W*” (p. 378).^3^ By the same logic, because *E* bears a constant relation to *V*_max_ (at least, during locomotion over a flat, solid support surface), information specifying *v*_front_*/E* also provides information about *v*_front_*/V*_max_. Of course, the relation between *E* and *V*_max_ would have to be learned through active exploration, just as the relation between *E* and *W* must be learned in the case of perceiving aperture size. As in the previous example, the outcome of such learning is a properly calibrated perceptual system that allows for the perception of *v*_front_/*V*_max_ on the basis of information about *v*_front_*/E*.

Let us now return to the observer, who must decide whether to pass in front or pass behind the moving obstacle. By detecting information about *v*_front_*/E*, the observer can perceive the minimum speed needed to pass in front in relation to her maximum speed capabilities. If the optically specified *v*_front_*/V*_max_ is greater than 1.0, then it is not possible to pass in front. The observer can immediately perceive that attempting to pass in front is futile and potentially injurious, and that the appropriate action is to slow down and pass behind the obstacle. On the other hand, if *v*_front_*/V*_max_ is less than 1.0, then it is still within the observer's capabilities to pass in front. Further, *v*_front_/*V*_max_ also specifies the proportion of the observer's maximum speed capabilities that she would need to move to pass in front. Thus, the availability of information about *v*_front_*/V*_max_ makes it possible to choose actions in a way that takes one's locomotor capabilities into account. In the section titled *Perceiving Passability and Choosing Routes Around Moving Obstacles* below, I will summarize findings from a recent study that demonstrate observers' ability to perform this task.

### Is locomotion guided by object motion in observer coordinates or world coordinates?

When people move in the presence of other moving objects, the motion of the object can be described in a reference frame that moves with the observer (i.e., observer coordinates) or in a reference frame that remains fixed relative to the stationary environment (i.e., world coordinates). A fundamental difference between the BA model and the affordance-based model concerns the reference frame within which object motion is perceived. In this section, I will explain why the two models use different reference frames and discuss empirical evidence that bears upon this issue.

**Figure 4:**
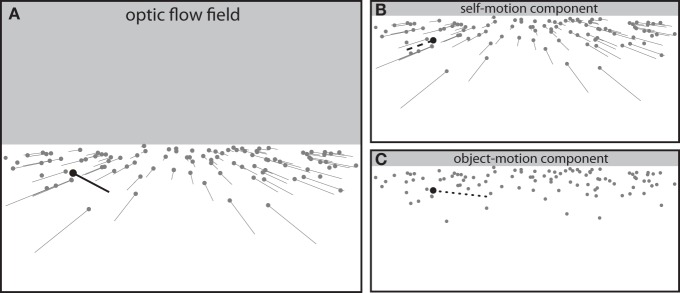
**(A)** Optic flow field generated by combined motion of observer and object (black dot). **(B)** The component of optic flow due to self-motion independent of object motion. **(C)** The component of optic flow due to object motion independent of self-motion. The optic flow field **(A)** is the vector sum of the self-motion **(B)** and object-motion **(C)** components.

Figure 4A depicts the optic flow field generated by an observer moving straight ahead with an object (black dot) moving from left to right across the observer's future path. The black vector represents the local optical motion of the moving object. Different combinations of observer motion and object motion with the same relative motion will result in identical local optical motion. This is because the local optical motion of the moving object is determined by the motion of the object relative to the motion of the observer—that is, object motion in a reference frame centered on the moving observer.

For an observer moving along a straight path, the lateral (i.e., side-to-side) optical motion of the moving object reflects the change in bearing angle^4^. For example, the rightward optical motion of the moving object in Figure 4A reflects a decrease in the bearing angle, specifying that the moving object is on course to pass in front of the observer. Conversely, if the moving object was drifting leftward in the optic flow field, the bearing angle would be increasing and the object would be on course to pass behind the observer. Thus, the change in bearing angle is reflected in the local optical motion of the moving object, which is determined by the relative motion between the object and the observer. As such, an observer using the BA strategy is guiding locomotion based upon object motion in observer coordinates.

In contrast, the affordance-based model relies on object motion perceived in world coordinates. This is because the properties upon which an observer using that strategy relies are independent of how the observer is moving at that instant. For example, for the observer in Figure 2A, the minimum speed needed to pass in front and the maximum speed needed to pass behind are the same regardless of how fast the observer is currently moving. These properties are defined in a reference frame that is fixed relative to the stationary environment rather than moving with the observer. Therefore, an observer using the affordance-based strategy is relying on object motion in world coordinates.

This raises an important question about the detection of information. If the optical motion of the object in the flow field reflects object motion in observer coordinates, then how can locomotion be guided by object motion in world coordinates? Formally, the optic flow field (Figure 4A) can be decomposed into two components: a self-motion component that reflects the motion of the observer independent of the motion of objects (Figure 4B) and an object-motion component that reflects the motion of objects independent of the motion of the observer (Figure 4C). The latter reflects object motion in a reference frame that is fixed in the world rather than moving with the observer—that is, object motion in world coordinates.

If locomotion is guided by object motion in world coordinates (as suggested by the affordance-based model), then the relevant information must be found, at least in part, in the object-motion component of optic flow. Indeed, looking back at Equation 7, one can see that the optical specification of the object's approach speed ${\dot{z}}_{m}$ involves ${\dot{\gamma }}_{m}$, which is the rate of change of γ due to the motion of the object independent of the motion of the observer. ${\dot{\gamma }}_{m}$ is effectively the downward optical motion of the base of the moving object in the object-motion component of optic flow. Thus, to detect the information that is needed to use the affordance-based strategy, observers must be capable of recovering the component of optic flow that is due to object motion.

#### Recovering object motion during self-motion

Formally, the object-motion component can be recovered from the optic flow field by factoring out the influence of self-motion, such that the component that remains reflects the optical motion due to object motion alone. One solution to this problem is to use global optic flow, which is a powerful source of information about the direction and speed of self-motion (Israël and Warren, 2005). By this account, the component of the object's optical motion that is due to self-motion is determined by global optic flow and factored out by the visual system, leaving the component that is due to object motion. Rushton and Warren (2005; Warren and Rushton, 2007, 2009) coined the term *flow parsing* to describe this process. In a series of psychophysical studies, they and other researchers (e.g., Matsumiya and Ando, 2009) have demonstrated that observers are capable of using global optic flow from the stationary background to recover object motion in world coordinates.

Of course, global optic flow is not the only source of information about self-motion. When self-motion is real and actively generated, non-visual information, which is known to play a role in the perception of self-motion (see Israël and Warren, 2005 for a review) is also available. In principle, the component of the object's optical motion that is due to self-motion (i.e., the component that must be factored out) can also be determined by non-visual information. Indeed, non-visual information about the speed (Fajen and Matthis, 2011) and direction (Fajen et al., in press) of self-motion also plays a role in recovering object motion in world coordinates. These findings and those of other researchers (e.g., Dyde and Harris, 2008; Calabro et al., 2011; MacNeilage et al., 2012; Warren et al., 2012) highlight the multisensory nature of the flow parsing problem.

A recent attempt to understand the neural substrates for this process revealed two clusters, one that includes the lateral occipital area, V3A, the kinetic occipital area, and human MT, and another that includes the ventral intraparietal sulcus and dorsal intrapariental sulcus medial (Calabro and Vaina, 2012). Activation in these areas was significantly correlated with performance on a psychophysical task involving object motion detection during simulated self-motion. It has also been proposed that neurons in MSTd and VIP that respond maximally to optic flow and vestibular signals in opposite directions could play a role in the recovery of object motion (Takahashi et al., 2007; Gu et al., 2008; MacNeilage et al., 2012). These cells might be ideally suited to detect object motion during self-motion because such situations result in local optical motion that does not match what would be expected based on vestibular input.

#### Flow parsing and visual control

So far, I have discussed the ability of human observers to use self-motion information to recover object motion in world coordinates. But does this process actually play a role in the visual guidance of locomotion during interception or obstacle avoidance? One way to test this hypothesis is by manipulating self-motion information in a virtual environment (VE) while subjects are performing an interception or obstacle avoidance task. For example, Fajen and Matthis (2013) instructed subjects to walk through a VE and choose whether they would pass in front of or behind an object moving across their future path, as in Figure 2A. They manipulated information about subjects' speed of self-motion by increasing the visual gain in the VE; that is, by translating subjects 50% faster through the VE compared to the real world. Importantly, the visual gain manipulation affected subjects' movement relative to the background only and not relative to the moving object. Thus, the local optical motion of the moving object was unaffected by the visual gain manipulation. If observers rely on object motion in world coordinates, and if global optic flow is used to recover object motion in world coordinates, then subjects should be less likely to perceive that they can pass in front when visual gain is increased. The results were consistent with this prediction. Similarly, route decisions were affected by manipulations of visual information about the direction of self-motion. In another study, the trajectories that subjects took to intercept a moving target were affected by manipulations of visual self-motion information (Fajen and Warren, 2004).

To summarize, effects of manipulations of self-motion information on behavior during interception and obstacle avoidance have been reported in several studies. These effects are predicted by the affordance-based model because locomotion is guided by information in the object-motion component of optic flow, and self-motion information is needed to recover that information. The BA model, on the other hand, does not account for these effects because locomotion is believed to be coupled to the local optical motion of the moving object in the optic flow field. Global optic flow and other sources of self-motion information do not play a role in the BA model. Therefore, manipulations of self-motion information that affect behavior would have to be interpreted as a reflection of some additional mechanism that interferes with the BA strategy. Insofar as the affordance-based model portrays these background flow effects as a reflection of an adaptive process rather than a flaw in the system, one could argue that it offers the more parsimonious account.

The broader issue raised in this section concerns the reference frame within which object motion is perceived during interception and obstacle avoidance. Whereas the BA model asserts that object motion is perceived in observer coordinates, the affordance-based model maintains that object motion is perceived in world coordinates. This entails the ability to recover the object-motion component of optic flow during self-motion, which is achieved using a combination of visual and non-visual self-motion information.

### Generalizing to arbitrary directions

The section titled *Body-Scaled Information* above described how the range of speeds needed to avoid a moving obstacle can be perceived based on information about *v*_front_ and *v*_behind_ (i.e., *v* < *v*_behind_ or *v* > *v*_front_). Of course, if the object is a target rather than an obstacle, the same information specifies the range of speeds required to intercept the target (i.e., *v*_behind_ < *v* < *v*_front_). Next, I will show that *v*_front_ and *v*_behind_ are specified not only for locomotion along the z-axis (as in Figure 2) but for any arbitrary direction. In the example in Figure 2, *v*_front_ and *v*_behind_ are specified for locomotion along the z-axis because the reference direction against which the visual angles α (corresponding to the visual direction of the object) and *γ* (corresponding to the angular declination) is measured is aligned with the z-axis. However, if α and *γ* are measured against some other arbitrary reference direction, then the optically specified required speed tells the observer how fast to move to intercept the target by moving in that direction. Thus, by varying the reference direction against which the visual angle α and *γ* are measured, such information could be used to perceive the speed needed to intercept a moving target by moving in any arbitrary direction.

**Figure 5:**
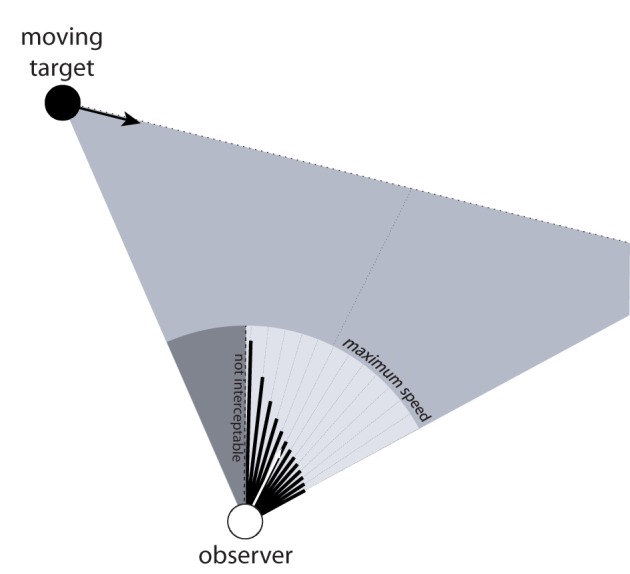
**Moving observer and moving target.** The black lines emanating from observer represent the optically specified speed required to intercept the moving target by moving in the corresponding direction. The light gray circular sector corresponds to the observer's maximum speed and the dark gray sector corresponds to directions for which interception is not possible because required speed exceeds maximum speed.

Figure 5 shows a moving observer and a target moving from left to right. The black lines emanating from the observer represent the optically specified speed needed to intercept the target by moving in that direction^5^. In particular, the lengths of the lines are proportional to the optically specified speed for each direction relative to the observer's maximum speed, which is represented by the light gray circle surrounding the observer. This illustrates that there is information about how fast to move to intercept the target not only for the current direction of locomotion but for any possible direction. Further, by calibrating that information to *V*_max_, one can also perceive the range of directions for which interception is not possible because the speed needed to intercept the target exceeds his or her maximum possible locomotor speed. In the section titled *Coordinating Speed and Direction of Locomotion during Interception* below, I will explain how such information can be used to coordinate speed and direction during interception, which addresses Limitation #3 of the BA model.

### Summary

To summarize, the affordance-based model offers an alternative to the BA model that can address the four limitations listed in the section *The Bearing Angle Model*. Unlike the BA model, the affordance-based model can explain how observers take object and body size into account, take their own locomotor capabilities into account, and coordinate speed and direction of locomotion. The model also explains why manipulations of self-motion information affect behavior during interception and obstacle avoidance.

## Scaling up to complex tasks

Having introduced the basic components and features of the affordance-based model, I am now ready to illustrate how this model can be applied to a wider range of problems that are encountered when guiding locomotion in complex dynamic environments.

### Perceiving passability and choosing routes around moving obstacles

Figure 2A depicts an observer moving along a straight path and an obstacle moving from left to right across the observer's future path. In such situations, the decision about whether to pass in front of or behind the moving obstacle must be made in a way that takes into account the size of the observer and the obstacle as well as the observer's locomotor capabilities.

Let us consider how the BA model and the affordance-based model perform on this task. As explained above, the BA model treats objects and the observer as points without physical size, which means that this model, at least in its original form, fails to capture how people take object and observer size into account. A potential solution to this problem is to treat the direction that would null the change in bearing angle as a repellor of heading and weight the strength of repulsion by a term that exponentially decays with obstacle distance (Cohen et al., 2006). The parameter of this term can be tuned to ensure that the observer veers far enough away from the obstacle to ensure collision avoidance. However, the parameter value is specific to the size of the observer and the size of the obstacle. Thus, when the environment contains multiple obstacles of varying sizes, the trajectories generated by this strategy may lead to collisions with large obstacles and veer unnecessarily far away from small obstacles.

By comparison, the affordance-based model capitalizes on information that reliably specifies required locomotor speed across variations in obstacle size. This was illustrated in Figures 3C,D, which show how the optically specified minimum speed to pass in front and maximum speed to pass behind systematically vary with obstacle size. Similarly, an observer who is properly calibrated to the relation between eyeheight and body size can perceive these properties in a way that takes body size into account. Thus, the affordance-based model is better suited to explain how people take the sizes of their body and obstacles into account.

The decision about whether to pass in front or behind the moving obstacle must also be made in a way that takes into account one's locomotor capabilities. Recall that the BA model ignores the fact that there are limits to how fast a person is capable of moving (see Limitation #2). Nonetheless, a proponent of the BA model could argue that the BA strategy could still be used to perceive whether it is possible to pass in front or behind. If the bearing angle is expanding, then the observer's current speed is sufficient to pass in front. Therefore, as long as current speed can be maintained, it is within the observer's capabilities to pass in front. Likewise, if the observer is moving as fast as possible and the bearing angle is shrinking, then the observer's maximum speed is not sufficient and it is not within his or her capabilities to pass in front. Thus, an expanding bearing angle specifies that it is possible to pass in front and a contracting bearing angle when the observer is moving as fast as possible specifies that it is not possible to pass in front^6^.

In other situations, the change in bearing angle is not informative about passability. For example, if the observer in Figure 2A was stationary rather than moving, then the bearing angle would be shrinking. This specifies that the observer's current speed (which is zero when the observer is stationary) is not sufficient to pass in front. However, depending on the object's trajectory and how fast the observer is capable of moving, the observer may or may not be capable of passing in front. The change in bearing angle provides no information about whether or not it is within the observer's capabilities to pass in front.

This leads to a testable prediction. If observers rely on the BA strategy to perceive whether it is within their capabilities to pass in front of an obstacle, then their ability to perceive passability should be impaired when they are not moving. This prediction is not supported. Observers are capable of accurately perceiving their ability to pass in front of a moving obstacle regardless of whether they are stationary or moving (Fajen et al., 2011).

The affordance-based model relies on information that specifies how fast the observer needs to move to pass in front in relation to how fast the observer is capable of moving. Such information can be used to perceive whether it is within the observer's capabilities to pass in front, and if so, the percentage of one's maximum speed that would be needed to pass in front (see the section titled *Taking Locomotor Capabilities into Account* above). Furthermore, this information is available regardless of whether the observer is stationary or moving, and therefore better accounts for the range of conditions across which observers can perceive which actions are within their locomotor capabilities.

### Perceiving passable openings between pairs of moving objects

**Figure 6:**
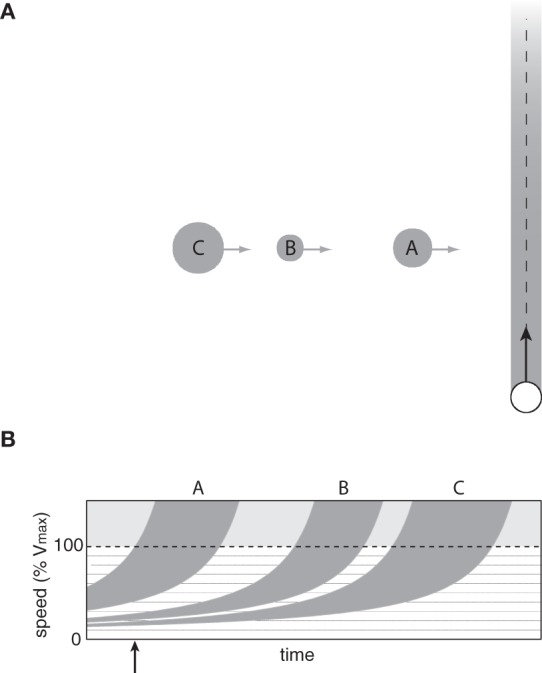
**(A)** Observer passing through a lane of obstacles on course to cross the observer's future path. **(B)** Optically specified range of speeds that would result in a collision with each obstacle as a function of time.

Having demonstrated the basic principles of the affordance-based model using a single moving obstacle task, we are now ready to consider situations involving multiple moving obstacles. Consider the task of passing through a lane of pedestrian traffic (see Figure 6A), which one might encounter at an intersection in a crowded airport terminal or shopping mall. In previous studies on passing through a lane of traffic, it was common to treat gaps between obstacles as targets to be intercepted (Chihak et al., 2010; Louveton et al., 2012a,b). By characterizing the situation as a target interception task, the BA model can be considered a possible strategy for regulating approach speed to an intersection.

However, the BA model does not explain how observers choose which gap to pass through in the first place. This is not a trivial problem because the passability of each gap depends on multiple factors, including the observer's body size, his or her locomotor capabilities, the spatial separation between obstacles, the speed of the obstacles, and the distance to the lane of traffic. Therefore, the decision about which gap to pass through must be made in a way that takes these factors into account.

The affordance-based model offers a starting point for understanding how observers choose appropriate gaps in these situations. The information described in the previous section specifies, for each obstacle, the range of speeds that would result in a collision between some part of the observer's body and some part of the obstacle. To illustrate this point, Figure 6B shows the optically specified range of collision speeds as a function of time for the situation depicted in Figure 6A (assuming that the observer remains stationary). By detecting this information, observers can perceive which pairs of obstacles form passable gaps and the range of speeds needed to safely pass through those gaps. For example, at the time indicated by the vertical arrow in Figure 6B, it is not within the observer's capabilities to pass in front of obstacle A because he or she would have to exceed 100% of *V*_max_. The observer could, however, safely pass between obstacles A and B by moving between 30 and 40% of *V*_max_. There is also a very narrow range of speeds (~19–21% of *V*_max_) that would allow the observer to pass between obstacles B and C. The important point is that the available information allows observers to perceive which gaps are passable and how fast to move to pass through those gaps. As such, the affordance-based model provides a basis for understanding how observers choose which gaps to pass through.

### Crossing multiple lanes of traffic

**Figure 7:**
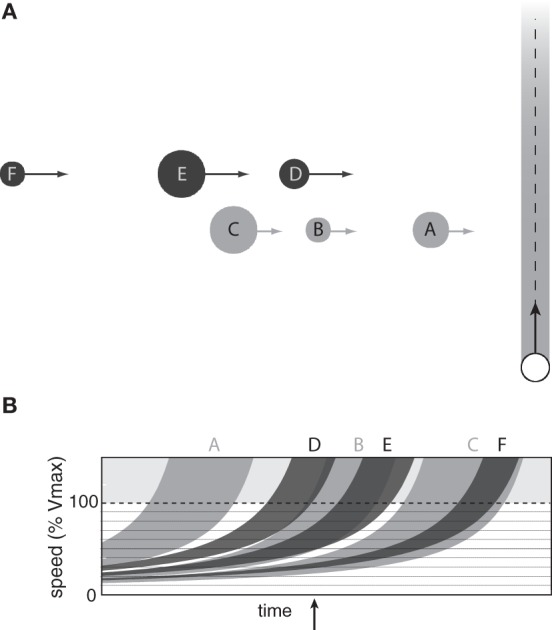
**(A)** Observer passing through two lanes of obstacles moving at different speeds. **(B)** Optically specified range of speeds that would result in a collision with each obstacle in the near (light gray) and far (dark gray) lanes as a function of time.

In many real-world situations, such as when crossing two or more lanes of vehicle or pedestrian traffic, reaching the goal requires passing through multiple moving gaps at different depths (Figure 7A). Under such conditions, it may be necessary to choose a gap in the first lane that takes into account the size and movement of gaps in subsequent lanes (Grechkin et al., 2012).

The affordance-based model can be easily generalized to situations in which there are multiple lanes of traffic at different depths. Each curve in Figure 7B shows the optically specified range of speeds that would result in a collision with one of the obstacles in Figure 7A. Light gray curves correspond to obstacles in the nearby lane and dark gray curves correspond to obstacles in the distant lane. As in the single-lane situation, the white spaces represent speeds at which the observer could move to safely avoid a collision. For example, at the time indicated by the vertical arrow in Figure 7B, the information specifies that it is not possible to pass between obstacles D and E in the far lane without colliding with obstacle B in the near lane. However, the observer could pass between obstacles B and C and then between obstacles E and F by moving between 40 and 50% of *V*_max_. Thus, the problem of choosing pairs of gaps to pass through when crossing multiple lanes of traffic can be solved by detecting information about *v*_front_ and *v*_behind_ for each obstacle.

### Coordinating speed and direction of locomotion during interception

When people intercept moving targets, there are an infinite number of combinations of locomotor speed and direction that would result in a successful interception. One of the strengths of the affordance-based model is that, unlike the BA model, it provides a basis for understanding how observers choose one combination of locomotor speed and direction from among all possible combinations. This is because the speed needed to intercept the target is optically specified not only for the current direction of locomotion but for any arbitrary direction (see the section titled *Generalizing to Arbitrary Directions* and Figure 5). Thus, if the observer wanted to intercept the target by moving at his or her preferred walking speed, then he or she could simply walk in the direction in which the optically specified required speed is equal to the preferred speed. Alternatively, if the observer wanted to intercept the target as quickly as possible, he or she could walk in the direction in which the optically specified speed is equal to his or her maximum locomotor speed.

### Simultaneous interception and obstacle avoidance

**Figure 8:**
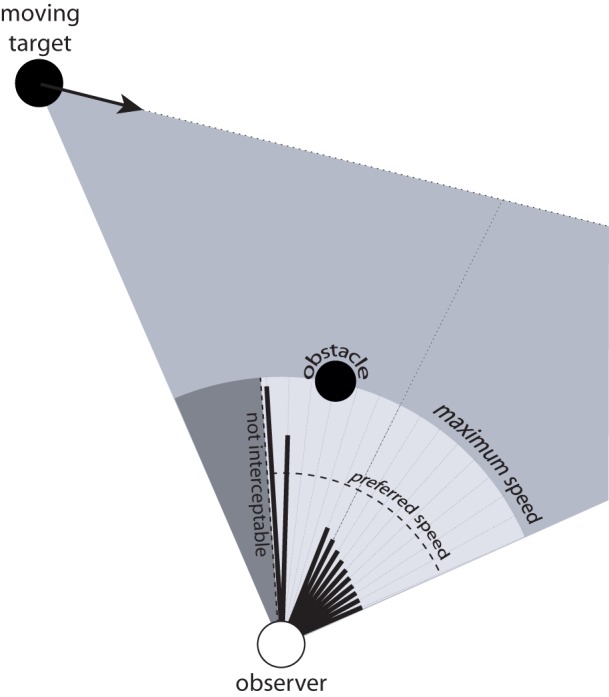
**Simultaneous interception of a moving target and avoidance of a stationary obstacle.** Black lines emanating from observer indicate speed required to intercept moving target in each direction. Directions that would result in a collision with the obstacle are removed.

The ability to perceive the speed needed to intercept a moving target as a function of direction could also allow people to choose appropriate routes when simultaneously avoiding a stationary obstacle. Figure 8 depicts a situation in which an observer must decide whether to pass to the right or left of a stationary obstacle while intercepting a moving target. As in Figure 5, the black lines emanating from the observer represent the speed needed to intercept the moving target for a range of directions. The lines that point in directions that would result in a collision with the obstacle are removed to indicate that these are not candidate directions of safe locomotion. Note that in the example in Figure 8, the available information specifies that the observer cannot intercept the target by moving at his or her preferred speed because doing so would result in a collision with the obstacle. In such situations, the observer can follow the route to the left of the obstacle and walk faster than normal to intercept the target, or follow the route to the right of the obstacle and walk slower than normal. One strategy for selecting routes in this situation is to choose the direction in which the optically specified required speed is closest to the preferred speed. The important point is that there is information that is available that allows the observer to choose which route to follow and to do so in a way that takes into account the speed that would be required to intercept the target for each possible route.

### Knowing when to abandon the chase

Another limitation of the BA model that was not mentioned in the section titled *Limitations of the BA Model* is that it offers no account of how an observer knows when to abandon the chase. For interception tasks, knowing when to give up is an extremely important aspect of achieving energetic efficiency. In the wild, for example, animals that do not know when to give up may expend more energy hunting than they consume by eating their kill. Indeed, one of the skills that cheetah acquire when learning to hunt is knowing when to stop running. This was demonstrated in a study by Caro (1994), who found that when adolescent cheetah abandon a chase during hunting, they travel an average of 18 m before doing so. By comparison, adult cheetah travel an average of just 2 m before giving up.

**Figure 9:**
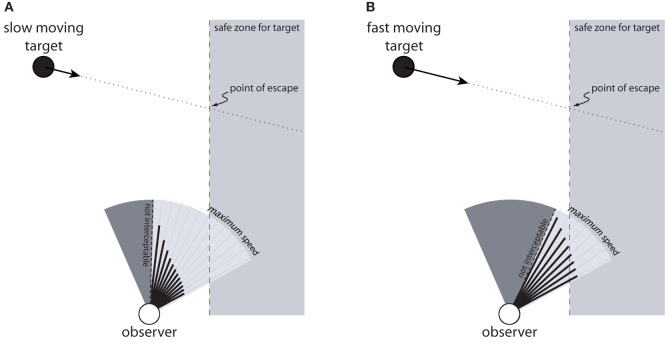
**Observer attempting to intercept a moving target before it reaches a safe zone (gray region on right side).** The observer is capable of intercepting the target in **(A)** but not in **(B)**.

The affordance-based model offers a possible account of how observers can perceive whether to continue chasing or to give up. If the information specifies that the speed needed to intercept the target is greater than maximum speed for all possible directions of locomotion, then the observer can perceive that the target is not interceptable. Attempting to chase the target in such situations would be a waste of energy. This solution even generalizes to situations in which the moving target must be intercepted before it reaches a safe zone. For example, suppose the observer in Figure 9 is a football player attempting to reach a ball before it rolls out of bounds, or a predator attempting to catch its prey before it reaches an area from which it can easily escape (e.g., a forest or a river). In Figure 9A, which shows a slow moving target, there are directions in which the observer could move to intercept the target before it reaches the safe zone. In Figure 9B, the speed required to intercept the target in any given direction is greater because the target is moving faster. As such, the observer would have to move faster than his or her maximum speed to intercept the target before it reaches the safe zone. Because the available information specifies the speed needed to intercept the target in each possible direction and in relation to maximum speed, the observer can perceive whether it is (Figure 9A) or is not (Figure 9B) within its capabilities to reach the target before it escapes. Such information could be the basis for deciding whether to attempt to intercept the target or stop moving and let the target go.

## Future directions

Although the affordance-based model captures important aspects of interception and obstacle avoidance that the BA model does not capture, there are questions about these tasks that remain open. First, in its current form, the model is unconstrained in that it does not make any specific predictions about which action the observer will select or which trajectory the observer will follow to intercept or avoid a moving object. For example, for the task of crossing a lane of traffic, the model describes how observers perceive which gaps are passable and the range of speeds needed to pass through each gap, but does not offer an account of which gap the observer will actually select. Similarly, when intercepting a moving target, the model describes how observers perceive the speed needed to intercept the target for each possible direction, but does not make any predictions about which direction the observer will actually follow to reach the target. In both cases, what is needed to generate specific predictions are additional constraints that presumably come from the observer's goal and intentions. For example, if the observer is attempting to move as closely as possible to his or her comfortable locomotor speed, the prediction in the case of crossing a lane of traffic would be the gap that allows the observer to move as closely as possible to that speed. Alternatively, if the observer is attempting to perform the task as quickly as possible, the prediction would be the gap that allows the observer to move as close as possible to his or her maximum speed. Additional theoretical development and empirical research is needed to understand how goals and intentions, which vary across observers and from situation to situation, constrain behavior on these tasks.

Another outstanding question concerns the limits on observers' ability to simultaneously make use of the available information for multiple obstacles. For example, in the situation depicted in Figure 7A involving multiple lanes of pedestrian traffic, the available information specifies which pairs of gaps are passable. However, perceiving passability for multiple gaps requires simultaneously detecting the relevant information for each obstacle in the scene. More research is needed to understand the limits of observers' ability to detect this information in environments with multiple moving objects.

## Conclusions

Few tasks exemplify the tight coupling of perception and action better than visually guided interception and obstacle avoidance. As such, information-based solutions such as the bearing angle model have been favored by researchers and for good reasons. Equally important, however, is the ability to choose actions and guide locomotion in a way that takes one's body dimensions and dynamics into account. No theory of visually guided locomotion would be complete without an account of how this is achieved.

In this article, I put forth a new affordance-based approach that attempts to do justice to the importance of taking one's body dimensions and dynamics into account. I showed how the affordance-based model accounts for the effects of self-motion information that have been reported in several studies, and demonstrated how this approach can account for a variety of complex behaviors involving moving objects.

The affordance-based model leads to important new insights into the components of the brain-body-environment system that make it possible for humans to perform these tasks: (1) the availability of eyeheight-scaled information that specifies dimensions of the environment in relation to dimensions of the body, (2) the ability of the perceptual system to calibrate itself to the relation between dimensions of the body (e.g., eyeheight and body width), and (3) the ability to recover object motion in world coordinates during self-motion. The latter insight, in particular, provides much needed justification for current and future research on the mechanisms involved in the perception of object motion by moving observers (Pauwels et al., 2010; Calabro and Vaina, 2012).

In a recent review paper, Warren (2007) observed that the visual control of action is turning out to be more interesting than many of the original models suggest. Although he was referring to models of locomotion over rough terrain, his observation applies to models of visually guided interception and obstacle avoidance as well. The affordance-based model may lack the simplicity of the bearing angle model. But what it lacks in simplicity it more than makes up in capturing adaptive behavior in complex dynamic environments.

## Funding

This research was supported by a grant from the National Institutes of Health (1R01EY019317).

### Conflict of interest statement

The author declares that the research was conducted in the absence of any commercial or financial relationships that could be construed as a potential conflict of interest.

## Footnotes

^1^Note that the point of reference on the ground plane for the angle γ is not the base of the object but rather the projection of the base of the object along the z-axis, as shown in Figure 2B. Although there may not be an identifiable point at this location, the tangent of γ is equal to the tangent of the angular declination of the base of the object divided by the cosine of the angular azimuth of the object (i.e., α in Figure 2B), both of which are optical variables.

^2^Equation 4 was adapted from Equation 2b in Bootsma and Craig (2002), with one minor modification. In Bootsma and Craig, TTC corresponds to the amount of time remaining until the center of the object crosses the z-axis. Therefore, their optical specification uses the angle between the z-axis and the center of the object, which they designated by θ. Because the affordance-based model requires the amount of time remaining until the leading edge of the object crosses the z-axis, Equation 4 uses the angle between the z-axis and the leading edge of the object (i.e., α in Figure 2B).

^3^Warren and Whang (1987) used *S* rather than *W* for shoulder width. I substituted W for S in the quotation to be consistent with the notation used in the present paper.

^4^When the observer is moving along a curved path, there is a component of the object's optical motion that is due to observer rotation. Because the bearing angle is measured against a reference direction that remains fixed in exocentric coordinates (see Figure 1), the influence of observer rotation must be factored out (Fajen and Warren, 2007).

^5^These values were calculated in Matlab using Equations 7 and 8, with the reference direction varying in 5° increments.

^6^This is similar to the strategy originally proposed by Oudejans, Michaels, Bakker, & Dolné (Oudejans et al., 1996) to explain how baseball outfielders perceive whether a fly ball is catchable.

## Appendix

The distance in Equation 5, $\left[z_{m}\text{​}\left(t^{*}\right)-z_{o}\text{​}(t)\right]$, is equal to the difference between the distance along the z-axis from the observer to the obstacle at time *t* and the change in the position of the obstacle along the z-axis between *t* and *t*^*^; that is:

(A1) $$\left[z_{m}\text{​}\left(t^{*}\right)-z_{o}\text{​}(t)\right]=\left[z_{m}(t)-z_{o}(t)\right]-\left[z_{m}(t)-z_{m}\text{​}\left(t^{*}\right)\right]$$

The change in obstacle position along the z-axis between *t* and *t*^*^ is:

(A2) $$\left[z_{m}(t)-z_{m}\text{​}\left(t^{*}\right)\right]=-{\dot{z}}_{m}\times TTC\times \left[1-\frac{W}{2x_{m\;-\text{ lead}}(t)}\right]$$

$-{\dot{z}}_{m}\times \text{TTC}$ is the distance that the obstacle moves along the z-axis between *t* and the time at which the leading edge of the obstacle reaches the z-axis. Multiplying $-{\dot{z}}_{m}\times \text{TTC}$ by $\left[1-\frac{W}{2x_{m-\text{lead}}(t)}\right]$ gives us the distance that the obstacle moves from *t* to *t*^*^. Next, we substitute Equation A2 into Equation A1 and Equation A1 into Equation 5, which yields:

(A3) $$d_{\text{front}}(t)=\left[z_{m}(t)-z_{o}(t)\right]+{\dot{z}}_{m}\times TTC\times \left[1-\frac{W}{2x_{m\;-\text{ lead}}(t)}\right]$$

Lastly, we substitute 2× *k* × *E* for *W*, which allows *x_m−__lead_* to be expressed in units of *E*, and divide both sides by *E*, which gives us Equation 6.
